# Glucocorticoids and cognitive function: a walkthrough in endogenous and exogenous alterations

**DOI:** 10.1007/s40618-023-02091-7

**Published:** 2023-04-14

**Authors:** D. De Alcubierre, D. Ferrari, G. Mauro, A. M. Isidori, J. W. Tomlinson, R. Pofi

**Affiliations:** 1 Department of Experimental Medicine, Sapienza University of Rome, Rome, Italy; 2 Department of Physiology and Pharmacology, Sapienza University of Rome, Rome, Italy; 3 Oxford Centre for Diabetes, Endocrinology and Metabolism, NIHR Oxford Biomedical Research Centre, University of Oxford, Churchill Hospital, Oxford, UK

**Keywords:** Cognition, Brain, Mineralocorticoid receptor, Glucocorticoid receptor, Cushing syndrome, Adrenal insufficiency

## Abstract

**Purpose:**

The hypothalamic–pituitary–adrenal (HPA) axis exerts many actions on the central nervous system (CNS) aside from stress regulation. Glucocorticoids (GCs) play an important role in affecting several cognitive functions through the effects on both glucocorticoid (GR) and mineralocorticoid receptors (MR). In this review, we aim to unravel the spectrum of cognitive dysfunction secondary to derangement of circulating levels of endogenous and exogenous glucocorticoids.

**Methods:**

All relevant human prospective and retrospective studies published up to 2022 in PubMed reporting information on HPA disorders, GCs, and cognition were included.

**Results:**

Cognitive impairment is commonly found in GC-related disorders. The main brain areas affected are the hippocampus and pre-frontal cortex, with memory being the most affected domain. Disease duration, circadian rhythm disruption, circulating GCs levels, and unbalanced MR/GR activation are all risk factors for cognitive decline in these patients, albeit with conflicting data among different conditions. Lack of normalization of cognitive dysfunction after treatment is potentially attributable to GC-dependent structural brain alterations, which can persist even after long-term remission.

**Conclusion:**

The recognition of cognitive deficits in patients with GC-related disorders is challenging, often delayed, or mistaken. Prompt recognition and treatment of underlying disease may be important to avoid a long-lasting impact on GC-sensitive areas of the brain. However, the resolution of hormonal imbalance is not always followed by complete recovery, suggesting irreversible adverse effects on the CNS, for which there are no specific treatments. Further studies are needed to find the mechanisms involved, which may eventually be targeted for treatment strategies.

**Supplementary Information:**

The online version contains supplementary material available at 10.1007/s40618-023-02091-7.

## Introduction

The hypothalamic–pituitary–adrenal (HPA) axis exerts many actions on the central nervous system (CNS) aside from stress regulation. Indeed, corticotropin-releasing hormone (CRH) fibers in the paraventricular nucleus of the hypothalamus also project to the brain stem and non-hypophysiotropic CRH neurons are abundant elsewhere, primarily in brain areas involved in sensory information processing (i.e., insulate cortex, parabrachial and solitary tract nuclei), emotional processing (i.e., amygdala, substantia nigra, and cingulate cortex), autonomic nervous system regulation (i.e., locus coeruleus), motor control (i.e., insulate cortex, substantia nigra), and cognitive functioning (i.e., pre-frontal cortex, substantia nigra) [1]. CRH also modulates behavioral activities concerning anxiety, mood, arousal, locomotion, reward, and feeding [2, 3], and increases sympathetic activation. Many of the non-hypophysiotropic behavioral and autonomic functions of these peptides can be viewed as complementary to activation of the HPA axis in the maintenance of homeostasis under exposure to stress (e.g., immune, cardiac, gastrointestinal, and reproduction effects) [4]. Hyperactivity of the HPA axis is a common neuroendocrine finding in affective disorders [2, 5], and the activation of central CRH pathways is a critical neurobiological substrate of anxiety and depressive states [3, 6]. The normalization of HPA regulation is highly predictive of successful treatment for these conditions.

Glucocorticoid (GC) secretion is regulated via a negative feedback mechanism, similar to the other hormonal axes. However, severe neurogenic stress and a large amount of CRH secreted in response to various stimuli can break through the feedback inhibition mediated by GCs. A higher level of feedback control is exerted by GC-responsive neurons in the hippocampus that project in the hypothalamus, determining the set point of pituitary responsiveness to GCs [7].

In this review, we describe the physiology governing the interaction between GCs, mineralocorticoids (MCs), and cognitive function, with the aim of unraveling the spectrum of cognitive dysfunction in different HPA-axis derangements involving endogenous and exogenous GCs’ secretion patterns, such as Cushing’s syndrome (CS), Adrenal insufficiency (AI), Congenital Adrenal Hyperplasia (CAH), and exogenous GCs (eGCs) treatment (see Table 1).

**Table 1:** Summary of affected cognitive domains and related brain areas, along with MRI findings, during HPA-axis derangements

Disease	Affected cognitive domains	Affected brain areas	MRI findings	Details/comments
Adrenal insufficiency (AI)	Verbal memory and learning Executive functions (mental flexibility, attention, working memory)	Hippocampus and limbic system Pre-frontal cortex	Reduction in brain and intracranial volume Reduction in the surface area of the inferior parietal and cingulate cortex Reduction in the right lateral orbitofrontal cortex volume	Ultradian cortisol fluctuation, disruption of circadian rhythm, unbalanced MR/GR activation and sleep disturbances, can all affect cognition in AI The role of disease duration, as well as that of replacement dosing, is still a matter of debate
Cushing Syndrome (CS)	Verbal and visual intellectual skills, including memory and learning Executive functions (visuo-spatial abilities, attention, concentration, working memory, processing speed)	Hippocampus and limbic system Neo-frontal cortex Pre-frontal cortex	Hippocampal atrophy Global brain volume loss Cerebellar cortex and pre-frontal regions atrophy Microstructural changes in white/gray brain matter	A full recovery does not always occur and might be influenced by several parameters
Congenital adrenal hyperplasia (CAH)	Lower general intelligence and IQ Visual intellectual abilities (memory, perception) Executive functions (working memory, visuo-spatial abilities, selective attention)	Frontal and pre-frontal cortex Hippocampus	Reduction in whole brain volume (hippocampus, amygdala, subcortical structures and cerebellum) Microstructural changes in white/gray brain matter Increased CSF volume	Cognitive impairment often associates with disease severity Adrenal crises, hyperandrogenaemia, cortisol deficiency, and GC dose regimens are possible risk factors for cognitive decline
Exogenous glucocorticoids (eGCs)	Declarative memory, processing speed (Steroid dementia syndrome) Working memory	Hippocampus Pre-frontal cortex	Hippocampal and amygdala atrophy Reduced brain volume Decreased neuronal vitality Reduced blood flow and metabolism in memory-related brain regions	Age, type and timing of eGCs administration, as well as duration of eGCs exposure likely act as influencing factors Acute/chronic effects of eGCs on cognitive function and brain structures is still a matter of debate GWS might affect cognitive function’ recovery

*CSF* Cerebral Spinal Fluid, *eGCs* Exogenous Glucocorticoids, *GR* Glucocorticoid Receptor, *IQ* Intelligence Quotient, *MR* Mineralocorticoid Receptor, *HPA* hypothalamic–pituitary–adrenal, *GWS* glucocorticoid withdrawal syndrome

### Physiology of glucocorticoids in the brain

**Fig. 1:**
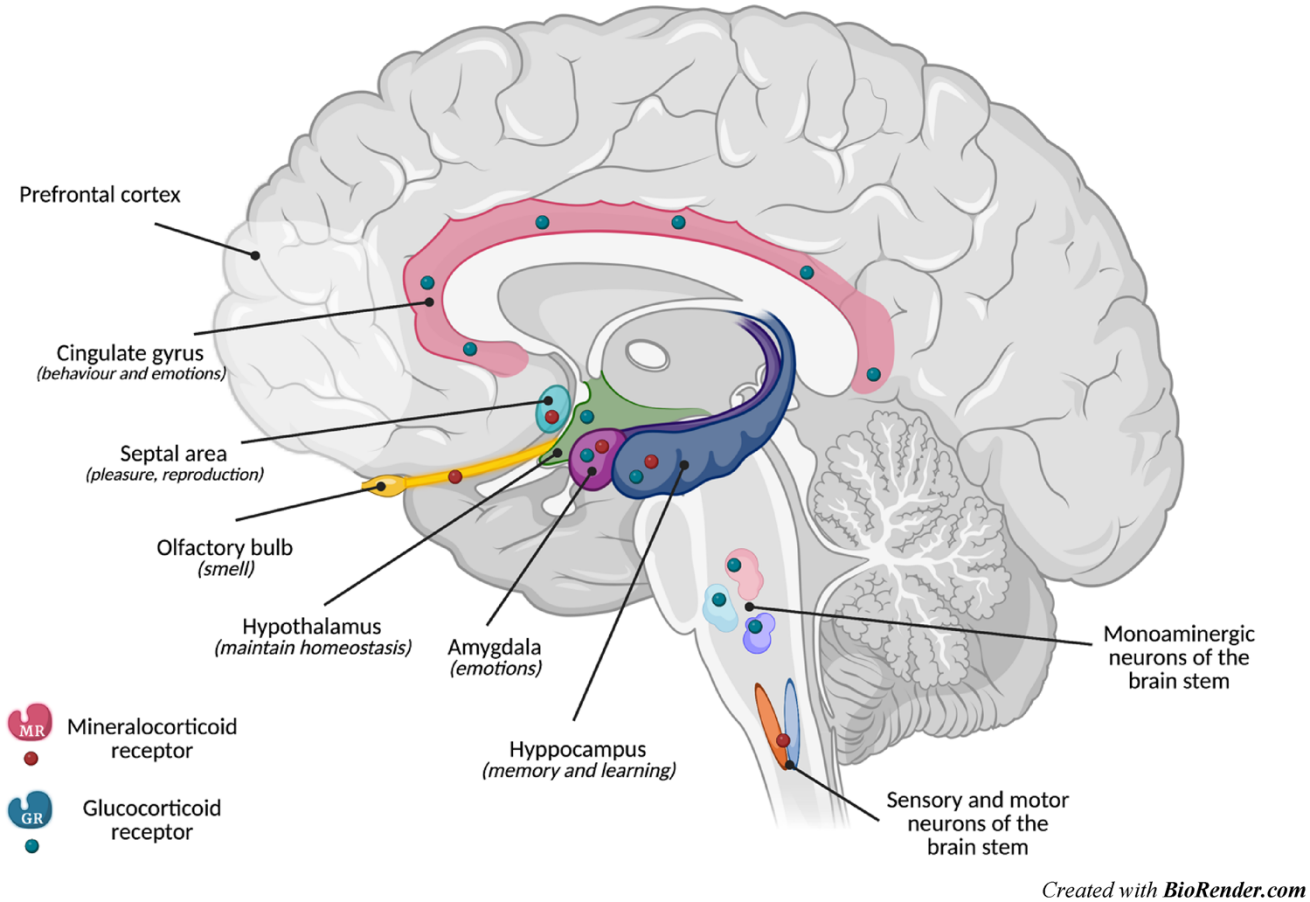
The spatial distribution of glucocorticoid and mineralocorticoid receptors in the brain. *Created with BioRender.com*

The kinetics of GC secretion follows two levels of control: a circadian rhythm, represented by extensive and pulsatile oscillations, on to which an ultradian rhythm is superimposed, interspersed within the daily kinetics with a recurrence of approximately one peak per hour, whose average amplitude influences the size of the major circadian fluctuations. Fluctuations in circulating GC correlate with the effects of hormones on target tissues. This pattern of GC secretion (rhythmic binding and dissociation of hormones from their receptors and pulsatility) is defined by the ultradian rhythm [8, 9]. Other than endogenous, eGCs, and other steroids can access the brain through the blood–brain barrier [10]. In the brain, GCs bind two different receptors: type I (the mineralocorticoid receptor—MR, so named because it binds aldosterone and GCs with high affinity) and type II (glucocorticoid receptor—GR, which has low affinity for MCs) [7, 11]. GRs are widely expressed throughout the brain, in neurons and glial cells, with high densities in limbic areas, monoaminergic neurons of the brain stem, and paraventricular and supraoptic nuclei of the hypothalamus, where they regulate the biosynthesis and release of vasopressin and CRH. The distribution of MRs is restricted to neurons of fewer brain areas: the limbic system (hippocampal formation, septal area, amygdala, and olfactory nucleus), sensory and motor neurons of brain stem and brain cortex [12]. The spatial distribution of GRs and MRs in the brain is summarized in Fig. 1.

While the GR has a high selectivity for GCs, MR is a promiscuous receptor capable of interacting with multiple ligands. Despite its high affinity for cortisol (tenfold higher compared to GRs [13]) and aldosterone, it can also bind progesterone, deoxycortisol, and deoxycorticosterone [13]. MR activation within the brain has been shown to mediate the stress-induced adaptive shift from a “cognitive” memory (mediated by hippocampus) to a more rigid, “habit-like” memory (mediated by striatum) [14, 15], reflecting the limited-resources condition of the “fight or flight” paradigm, in which the system is conceived to rapidly recall and enable simple stimulus responses to face a stressful situation.

The complex interaction between steroid hormones and the areas responsible for several cognitive domains within the brain is coordinated by specific enzymatic activities and enzyme/receptor zonation.

The ligand specificity of the MR, which determines its activation by either GCs or aldosterone in various tissues, is mediated by the 11-beta-hydroxysteroid dehydrogenase (11β-HSD) enzymes [16]. There are two different isoforms of 11β-HSD: type 1 and type 2. The type 1 enzyme is widely expressed in key GC target organs (adipose tissue, skeletal muscle, and liver), including the brain [17, 18]. It predominantly converts inactive cortisone to active cortisol [19], thus amplifying local GC bioavailability. Conversely, 11β-HSD type 2 inactivates cortisol to cortisone [20]. The primary role of 11β-HSD type 2 is to protect the MR from inappropriate activation by GCs [21] in mineralocorticoid sensitive tissue, such as the kidney, placenta, colon, and salivary glands by converting them in their 11-oxo metabolites, allowing aldosterone to bind to MR despite its 100–1000-fold lower concentration in the bloodstream, compared to GCs [22].

The different expression of these two isoforms within the brain areas impacts the activation of both MR and GR. Indeed, only 11β-HSD type 1 is expressed in hippocampal cells and limbic structures and, therefore, MRs are usually saturated by physiological cortisol concentrations in these areas, making them crucial for the emotional and memory processes [23]. Some brain areas also express 11β-HSD type 2, making them sensitive to aldosterone, such as the nucleus of solitary tract, which is implicated in modulating the behavioral response (including appetite, mood, and arousal) to fluctuations of sodium concentrations [24].

The regional distribution of MR and GR within the brain adds further complexity, suggesting that the brain response to GCs is extremely tightly regulated with a delicate between substrate availability and MR and/or GR activation. Specifically, during physiological basal and low pulsatile GCs conditions, GCs preferentially bind and activate the MR, resulting in an increased MR/GR activation ratio. This dynamic has a predominantly neuro-projective role. In fact, basal MR signaling contributes to the stabilization of excitatory postsynaptic currents, generating a negative feedback signal directed to the hypothalamus, limiting detrimental GC effects and mediating behavioral adaptation by proactively regulating the sensitivity of the neuroendocrine stress-response system [25]. Indeed, blocking MR activity with spironolactone has been shown to impair selective attention and visuo-spatial memory in healthy men [26], whereas MR stimulation with fludrocortisone improved spatial memory [27]. In contrast, when GC bioavailability increases beyond the saturating capacity of the MR receptors, as happens during the stress response, GCs bind to the otherwise inactive low-affinity GR, causing an inversion in the MR/GR ratio (i.e., a reduction of the MR/GR activation ratio) which allows the gradual recovery from stress during reactive mode [12]. However, chronic and prolonged stress may promote an excessive reduction of the MR:GR ratio [28, 29]. This results in the progressive loss of hippocampal ability to exert negative feedback on the HPA axis and phenomena of maladaptive synaptic plasticity. The effects of GR receptor activation on neurogenesis and the reduced neuroprotective MR signaling affect the reversibility of the functional disorder, precipitating the appearance of the pathological phenotype [30, 31].

In the CNS, gene expression regulation by GCs is most significant in the areas where neurogenesis occurs, even in adulthood. Neuronal proliferation and differentiation correlate inversely with the central bioavailability of GCs: in the presence of reduced concentrations of cortisol and, therefore, in conditions of activation of the MR receptors alone, higher growth and differentiation rates can be documented. However, excess central bioavailability of cortisol leads to GR activation and precipitates a reduction in neurogenesis [32]. Numerous pathological and para-physiological conditions can, by increasing cortisol levels, affect adult neurogenesis: among these, all the endocrine diseases causing alteration in GC secretion patterns. These might be responsible for an imbalance between GR and MR activation in the brain, leading to detrimental effects on cognitive function.

### Cushing’s syndrome and cognition

Cushing’s syndrome (CS) is a severe, chronic, and life-threatening disease caused by prolonged hypercortisolism, which can be endogenous or exogenous. Endogenous hypercortisolism can result from ACTH-secreting tumors—either pituitary (Cushing’s Disease, CD) or extra-pituitary (ectopic CS) [33]—or ACTH-independent increase in adrenal production due to bilateral gland hyperplasia or tumoral lesions [34]. Of note, moderate hyperactivity of the HPA axis can also derive from adrenal incidentalomas presenting with mild autonomous cortisol secretion (MACS) and non-neoplastic hypercortisolism (NNH) states (including chronic alcoholism, polycystic ovary syndrome, anorexia nervosa, and psychiatric disorders) [35], resulting in partial clinical and/or biochemical overlap with overt CS [36]. Long-standing exposure to GC excess cause multiple medical comorbidities, most notably metabolic syndrome, increased cardiovascular risk, immune and musculoskeletal disorders, subfertility, and dermatological manifestations [37]. Neuropsychiatric disorders are common in patients with CS, the most frequent being major depression (50–81%) and, to a lesser extent, anxiety (66%) and bipolar disorder (30%) [38]. Cognitive impairment (sometimes also called “steroid dementia syndrome”) is another common finding in patients with CS, which significantly impairs patients’ quality of life [39]. Its prevalence is extremely variable, ranging from 15 to 83% of cases [40, 41].

Cognitive decline and mood disorders often overlap in patients with CS [40]. A recent prospective study [42] demonstrated that the improvement in the cognitive impairment in patients with CD after trans-sphenoidal surgery parallels (and perhaps depends on) the improvement in depressive scores, thus highlighting the close relationship between mood disorders and cognition [42]. Indeed, 4 weeks of antidepressant therapy (such as serotonin re-uptake inhibitors) proved effective in improving cognitive function (e.g., memory, orientation, spatial navigation, and verbal fluency) in adolescents with CD [43].

However, cognitive impairment can often occur as a separate neurological disorder in CS [38, 44]. Memory is the domain that is most frequently affected (86% of cases) [45] due to the density in GR and lack of 11β-HSD type 2 activity in hippocampal neurons, making them highly sensitive to HPA-axis hyperactivity. Indeed, moderate memory impairment has been reported in patients with CD [46], regardless of associated neuropsychiatric comorbidities. Up to two-thirds of patients with active CS report difficulties with the registration of new information, forgetfulness for appointments and locations of objects, as well as shortened attention span, reduced concentration ability, and impaired comprehension abilities [45, 47, 48]. In addition, worse performance on visual and spatial information [49, 50], attention, executive functioning, and non-verbal aspects of memory [50] have also been described. Although the data are not all consistent [46], a direct relationship between ACTH and cortisol levels with the severity of neuropsychiatric impairment has been described [44, 51].

A recent meta-analysis [47] including 294 patients with CS also confirmed cognitive decline in seven out of eight cognitive domains. Memory and learning-related functions (both visual and verbal) were the most impaired, along with the general intelligence and language skills domains. Other deficits concerned executive and visuo-spatial functions, as well as attention and processing speed [47].

Recently, the possibility that even mild hypercortisolism might associate with impaired cognitive function has been questioned. A small multicentre study evaluating cognitive function in 23 patients with MACS found differences in some (verbal fluency, symbol coding, and executive function) but not all (verbal and working memory) cognitive domains [52]. However, very recently, a prospective study on 63 patients with adrenal incidentalomas described worse performances regarding working memory, visuo-spatial domains, and overall cognitive function in patients with MACS compared to those with non-functioning adrenal adenomas. Multivariate linear regression also showed post-DST cortisol as a risk factor for cognitive impairment [53]. Albeit conflicting, these preliminary findings suggest that mild hypercortisolism might affect mental health and cognitive status, although its impact has yet to be clearly investigated. Moreover, a direct comparison between patients with MACS and overt CS has never been performed, underlining the urgent need for further prospective studies to elucidate the contribution of hypercortisolism degree on cognitive impairment.

Interestingly, the pattern of cognitive deficits in CS is similar to that described with aging [54], with impairment in general intellective capacity and poorer performances in executive functions, spatial memory, and attention tasks. This suggests that cortisol excess might exhibit an “aging-like” effect, further exacerbating the cognitive impairment typical of older age [54]. Similarly, higher plasma cortisol levels have been linked to faster cognitive deterioration in patients with Alzheimer’s Disease, and researchers are questioning whether alterations in genes involved in the regulation of the GC system may influence the risk for this condition. The study showed that patients carrying the apoE4 allele (a known risk factor for Alzheimer’s Disease) have elevated cerebrospinal fluid cortisol levels [55], reinforcing the hypothesis that hypercortisolism accelerates hippocampal damage and leads to a dementia-like cognitive phenotype [56]. Moreover, a rare haplotype in the region of the gene encoding 11β-HSD has been found to confer a sixfold higher risk for sporadic Alzheimer’s Disease [57], likely increasing neuronal GC-associated neurotoxicity.

From a morpho-structural point of view, early magnetic resonance imaging (MRI) studies linked hypercortisolism and hippocampal atrophy in patients with CS [58]. Since then, hippocampal atrophy has been reported as the most common finding in patients with active CS [47, 59–61], although global brain volume loss [62] and atrophy [48, 59, 63], smaller volumes in the cerebellar cortex [64], and the pre-frontal regions have also been observed [65]. Recently, decreased hippocampal volume (HV) was described only in patients whose memory scores were impaired [48], suggesting that hippocampal atrophy likely reflects higher disease severity and a more advanced state of cognitive dysfunction. Therefore, MRI assessment of HV may underestimate the neurocognitive consequences of CS [66].

Aside from HV, a negative correlation between urinary-free cortisol and subcortical gray matter and cerebral white matter MRI intensity has been reported [67]. Changes in white/gray matter (consistency, intensity, and homogeneity), as well as axonal and myelin damage at MRI, might precede detectable changes in brain volume [67] in CS, as demonstrated in patients with both active and cured CS [68, 69]. Interestingly, these alterations associate with worse information processing speed [70] and overall cognitive performance scores [69]. Recently, the use of advanced MRI sequences [71] revealed specific microstructural changes involving hippocampus/parahippocampal areas, which correlates with the clinical severity of CD and the degree of cognitive impairments [71].

Notably, functional and structural alterations similar to those found in CS have also been identified in states of NNH. HPA-axis hyperactivation has been hypothesized to play a role in the “alcohol dementia”, the cognitive deficits observed during and after chronic alcohol consumption withdrawal [72]. The underlying mechanisms are not entirely clear, but, considering the global brain volume alterations frequently described during alcohol intake (mainly involving white frontal matter [72], white matter microstructure [73], and hippocampal volume [74]), a glucocorticoid-mediated toxicity at the hippocampal level has been suggested [75]. Among the main causes of NNH, major depressive disorder often presents cognitive dysfunction as a core feature of the clinical spectrum [76]: explicit memory and executive functions are the most affected cognitive domains, with the hippocampus being the main impaired brain area [77]. Similarly, anorexia nervosa has been linked with impaired cognition [78], decreased HV [79], and altered functional connectivity, especially in the corticolimbic circuit, which is deeply involved in cognitive control [80]. Notably, in these patients, serum cortisol levels are inversely related to hippocampal and gray matter volumes [79].

In patients with CS, a prompt diagnosis of cognitive dysfunction is crucial as clinical manifestations often precede brain anomalies detected by imaging [81]. Longer disease duration and older age are associated with limited recovery of brain functioning, whereas earlier diagnosis and rapid normalization of hypercortisolism appear to reduce the progression of brain damage and functional impairments [81]. According to a recent meta-analysis [47], the majority of impaired cognitive domains undergo a significant improvement following surgery. Similarly, medical treatment of hypercortisolism, either with GC-receptor antagonists or steroidogenesis inhibitors, can improve psychiatric symptoms within weeks of therapy [82–84].

The timing and degree of the reversibility of cognitive dysfunction in CS are still a matter of debate. Some studies have reported both cognitive and brain morphological improvement following treatment [85–89]. A recovery in verbal fluency/recall and HV increase has been documented 18 months after medical treatment, with younger age being a predictor for functional recovery [88]. Endorsing these data, middle-aged (< 60 years), comorbidity-free patients in long-term (10 years) remission from CD exhibited the same hippocampus and pre-frontal cortex-dependent memory functioning when compared to healthy controls. While these results should be interpreted with caution due to a potential selection bias, it could be argued that the persistence of cognitive impairment after CD remission could be partially attributable to other comorbidities that might potentially affect cognition (i.e., diabetes, age, cardiovascular diseases, hormonal disbalance, and psychiatric disorders), further highlighting the importance of shortening the exposure to hypercortisolism and its comorbidities to preserve cognitive functioning [90].

Nevertheless, several studies have suggested that cognitive function impairment might not completely resolve following surgical treatment of CS [91]. Persistent cognitive impairment (attention, spatial orienting, alerting, working memory, verbal fluency, reading speed [92], and trail-making [93]) has been described after long-term remission in CD [92, 94] and adrenal CS [92], without differences between different etiologies [92]. To date, no accurate predictors of cognitive impairment recovery following remission have been identified.

The extent of the reversibility of structural brain abnormalities in patients with CS is still a matter of debate. The reduction of brain and HV in patients with active CD has been described as partially reversible after cure in some [95], but not all studies [48], and the amelioration of hippocampal morphology has been associated with symptom improvements [96].

Similarly, in patients with NNH, the resolution of the primary *noxa* usually associates with partial improvement of cognitive functioning, although this is not often mirrored by restoration of physiological brain morphology [73, 79, 97].

Decreased cortical thickness [98] and smaller volumes in the anterior cingulate cortex [99] can persist after long-term CS remission, but an inverse correlation with disease duration suggests a direct link between the prolonged exposure to GC excess and the alterations of brain structures involved in emotional and cognitive processes [100]. Indeed, a recent study including young patients (< 32 years) with short disease duration (< 3 years) has demonstrated a rapid and complete recovery (within 3 months of surgical treatment) of brain volume loss observed in the active phase of the disease [101]. New research approaches using functional MRI spectroscopy [102] are being used to explore neuronal vitality markers within the hippocampal area and their possible role in reversing hippocampal alterations.

It must be noted that successful treatment of CS can be followed by iatrogenic adrenal insufficiency, which is often transitory and promptly replaced with GC therapy. Current literature assessing the reversibility of cognitive and brain structure abnormalities following CS treatment is rather heterogeneous, with studies including hypocortisolemic [91], eucortisolemic [65], or mixed [88] cohort of patients. Whether the post-treatment serum cortisol fluctuations might shape cognitive recovery remains largely underexplored.

In conclusion, cognitive impairment is a common finding in patients with active CS and is directly related to cortisol levels and the duration of hypercortisolism. Long-term exposure to elevated cortisol levels affects several cognitive domains, including memory, verbal intellectual skills, and learning, reflecting a solid hippocampus and neo-frontal cortex involvement. Lack of complete normalization of cognitive functioning after treatment is likely attributable to GC-dependent structural brain alterations, which, if present at diagnosis, generally persist even after long-term remission.

A summary of the current evidence for cognitive function in CS is shown in Table 1 and Supplemental Table 1.

### Adrenal insufficiency and cognition

Adrenal insufficiency (AI) is a relatively rare endocrine disorder with multiple causes that can be divided into primary (adrenal), secondary (pituitary), and tertiary (hypothalamus or eGC treatment) forms. Each form of AI has distinctive causes with implications for treatment and follow-up. Patients with primary AI require GC and MC replacement therapy, whereas individuals with secondary and tertiary AI usually necessitate only GC replacement [103–105]. If left untreated, AI is a life-threatening condition.

Patients with AI exhibit a broad spectrum of non-specific symptoms (e.g., fatigue, weakness, mental straining, and malaise) [106–108], and therefore, the diagnosis of psychiatric conditions is challenging, often delayed or mistaken [109].

Cortisol deficiency is known to induce many neuropsychiatric alterations, such as depression, delirium, and delusional ideas [110], that generally reverse after appropriate GC treatment. However, a few studies investigated cognitive function in patients with AI.

Compared to matched healthy controls, impaired declarative memory [111–114], poor performances in episodic memory [111], and verbal memory and learning [114] have been reported in patients with AI, despite stable replacement therapy. Worse performances in verbal and visual memory tasks [115], as well as some executive functions (including attention-related tasks [116] and processing speed) have also been observed, although some data are conflicting [113, 115, 117]. Conversely, no significant differences have been found with respect to concentration, working memory, and visuo-spatial functioning [111, 114].

The cognitive impairment found in AI patients is thought to be due to multiple cooperating pathogenic factors. A recent meta-analysis including more than 500 patients reported that both increased and decreased GCs levels might be responsible for impaired hippocampal-dependent memory and cognitive function [118]. In support of this hypothesis, the inhibition of cortisol production via metyrapone administration caused memory impairment in healthy patients, which was reversed after hydrocortisone treatment [119]. Furthermore, hypoadrenal patients who have experienced a long diagnostic delay and consequent prolonged exposure to cortisol deficiency have worse cognitive performance, notably declarative memory and processing speed [111]. Although there is some compelling evidence suggesting that circulating serum cortisol levels are an important contributor, the actual impact of cortisol deficiency has been questioned in a recent study, in which patients who omitted their morning hydrocortisone replacement doses did not report cognitive dysfunction [115].

In addition to absolute cortisol levels, both the circadian and ultradian rhythms have an important role to play [120]. Indeed, patients with AI generally experience an altered circadian profile of cortisol levels, ranging from low basal concentrations to supraphysiological peaks following acute GC administration. As already discussed, physiological cortisol levels influence cognitive function through a fine regulation of the dynamic balance between MR and GR activation within the brain. However, excessive cortisol fluctuations during replacement regimens might affect MR/GR activation ratio, leading to impaired cognition in patients with AI [121–123]. MR activation has been linked to the ability to learn new information, whereas GR activation is generally associated with memory storage and retrieval [121]. Albeit dedicated pharmacodynamics studies aiming to find the dose of MC able to elicit effects on cognitive functions have never been performed, there are multiple evidences (in healthy subjects as well as in patients with AI) demonstrating that fludrocortisone administration at doses equal or higher than those employed in clinical practice (0.1–0.4 mg per day) causes high MR occupation and improves different domains of memory tasks (verbal [124–126], working [126, 127], and visuo-spatial memory [128]). However, attention and executive functions were impaired during low MR occupation, even after a single omission of a fludrocortisone daily dose [124].

In this context, taking into account the impact of GCs on brain regions, such as the hippocampus and the pre-frontal cortex, the importance of using physiological total daily GC dose in a circadian fashion is crucial. This has to be taken into account both from a functional and structural point of view [129, 130], as degeneration of hippocampal neurons [131], and related impaired performance on declarative memory tasks [132] have been described in AI patients. Moreover, higher hydrocortisone replacement doses have been associated with worse cognitive function, mainly impacting attention, executive function, visual and motor tasks [133], as well as short-term memory [134]. Notably, these cognitive domains are closely linked to the hippocampus, a region exhibiting the highest density of GRs and MRs, and therefore particularly vulnerable to fluctuations in cortisol levels.

The role of disease duration has been proposed as a potential risk factor for cognitive dysfunction in AI, but studies have reported conflicting results and should be interpreted with caution due to the small sample size and follow-up duration. Longer disease duration that was associated with impaired verbal learning [115] and processing speed [111] and prolonged hydrocortisone treatment was found to negatively affect hippocampal structure and function [131]. However, these findings were not confirmed in later studies [133, 134] and require additional investigation.

A recent review of the literature underlined the concept that cognitive impairment is closely associated with sleep disturbances in patients with AI [122]. Hypoadrenalism is associated with poor sleep quality [135, 136], and sleep disturbances are reported in up to 34% of patients [135]. Sleep plays critical roles in memory consolidation [137, 138], a process starting during *slow wave sleep*, in which HPA-axis suppression allows the retainment of memories acquired throughout the day [139]. Cortisol ensures initiation and transition between different sleep stages [140–142]. Its physiological nadir occurs during the first hours of nocturnal sleep and allows predominant MR activation in the brain, which is crucial for the consolidation of declarative memories. It would be anticipated that any impairment in the normal circadian rhythm would be detrimental to this process [143]. For instance, night cortisol levels might be too low to activate the MR in patients with AI. Accordingly, impaired declarative memory retention associated with poor sleep quality has been described in patients with AI compared to healthy controls [144]. Despite stable replacement therapy, patients with AI often report reduced quality-of-life outcomes, notably related to sleep disturbances and other cognitive dysfunction (memory impairment and affective disorders among the others) [112, 135, 145].

Several studies have investigated the impact of different replacement regimens on sleep and cognitive functioning in this context. Albeit it generally fails to mimic normal circadian rhythm [146], HC replacement therapy ensures a more consolidated sleep pattern [147], as opposed to GC deprivation which results in poor quality of sleep [136, 147]. Higher HC doses at night, multiple daily HC administrations, and circadian rhythm disruption [117, 148] all lead to HPA-axis dysregulation and sub-optimal MR/GR activation [121], which in turn leads to poor sleep quality and cognitive impairment [149].

In conclusion, mild cognitive impairment is common in patients with AI, mainly affecting declarative memory, verbal learning, and processing speed. From a pathogenic perspective, there are multiple factors that have significant contributions to its development. Ultradian cortisol fluctuation, disruption of circadian rhythm, unbalanced MR/GR activation, and sleep disturbances can all affect cognition in AI [60]. The role of disease duration, as well as that of replacement dosing, is still a matter of debate.

A summary of the current evidence for cognitive function in AI is shown in Table 1 and Supplemental Table 2.

### Congenital adrenal hyperplasia and cognition

Congenital adrenal hyperplasia (CAH) includes a group of autosomal recessive disorders characterized by enzymatic defects in adrenal steroidogenesis [150]. Mutations involving the 21-hydroxylase gene account for 95% of cases [151]. According to the enzymatic defect, the impairment in GC production results in increased ACTH secretion, which leads to a shift of the steroidogenic pathway toward sex steroid production, with different clinical pictures [152].

CAH can be classified into different forms according to residual enzyme activity. The classic CAH is generally associated with a more severe phenotype. In the most severe Salt-Wasting (SW-CAH) form, there is little or no residual enzyme activity, whereas patients with the Simple-Virilizing (SV-CAH) form still retain 1–5% enzyme activity [153]. The non-classic form of CAH is associated with various degrees of enzyme activity and is characterized by a late onset and milder symptoms, such as female virilization, menstrual irregularities, and subfertility [154]. Treatment generally consists of GC with or without MC replacement therapy, aiming to decrease androgen secretion, correct cortisol deficiency, reduce virilization, and restore fertility [155].

Several studies have investigated cognitive function in patients with CAH describing lower Intelligence Quotient (IQ) with worse overall intelligence [156–160]. A recent study also reported worse performances in visual perception, visual memory, and executive functioning in patients with CAH compared to age-matched, healthy controls [161].

Interestingly, a more severe disease phenotype also associates with greater cognitive impairment. A Swedish epidemiological study reported SW-CAH patients as less prone to complete primary education, exhibiting a higher frequency of disability pensions and sick day leaves compared to controls [162]. Other studies demonstrated worse performance in several cognitive domains (visual memory, fluid intelligence, and non-verbal reasoning tasks) in SW [157, 163, 164] compared to SV-CAH patients [158].

Various factors have been implicated in the development of cognitive impairment in SW-CAH patients. Brain injury related to hyponatraemic episodes secondary to salt-wasting crises has been proposed among the possible underlying mechanisms for lower IQ [157, 165]. Later studies have confirmed this finding in patients with CAH with a positive history of adrenal crises [163, 164]. The contribution of androgen excess to cognitive impairment may also be significant. Patients with CAH (both male and female) are typically exposed to increased androgen levels in utero [166]. However, CAH boys compensate for this higher exposure by reducing testicular androgens, maintaining higher, but still acceptable testosterone levels [166]. On the contrary, CAH females are more susceptible to gestational androgen excess [167]. Increased pre- and post-natal androgen levels affect neuronal development and brain functional connectivity [168, 169], and impair sex-specific cognitive dimorphic abilities, finally leading to an increased risk of learning disabilities [159]. Neuronal myelinization [170, 171] and brain hemispheres maturation [164] are among the alteration described.

Of note, increased levels of precursors (17-OH progesterone, 21-deoxycortisol, and 21-deoxycorticosterone, as well as their metabolites) are commonly observed in CAH patients. These molecules bind to the MR with different affinities and influence their activity [172], possibly interfering with the MR/GR balance in the brain. However, their impact on cognitive functioning in CAH patients has yet to be studied.

Androgens excess can affect cognitive domains differentially in women with CAH. Better performances on tasks involving cognitive domains which typically favor males [173] (mental rotation, spatial perception [174], and fine motor skills [175]) have been described: female patients with CAH and severe disease (and therefore the highest level of in-utero androgen exposure) perform similarly to both healthy males and male patients with CAH regarding spatial cognition [176]. On the other hand, not all cognitive domains benefit from androgen over-exposure and several studies have reported worse short-term memory than in controls [157, 167, 177].

Interestingly, some authors described a lack of cognitive impairment in children and adolescents with CAH, as opposed to their older counterparts [156, 177, 178]. While this observation might reflect a genuine age-related difference exerted by the underlying disease, it is plausible that this discrepancy might relate to the long-term therapeutic (perhaps supraphysiological) exposure to GCs. As discussed above, GC treatment often fails to replicate physiological circadian rhythms [179], resulting in times of under- or over-treatment, both of which can negatively impact cognitive function [177], especially memory [180]. Recent observations have also reported significantly lower IQ in poorly controlled patients affected by SW-CAH, with multivariate analysis showing that in addition to androgen levels and hyponatraemic episodes, higher GC doses were associated with cognitive impairment [163]. Indeed, GC over-treatment is known to affect hippocampal development and function by altering neuronal structure [59], with hippocampal subfield CA1 (closely associated with learning and memory processes) [181, 182] displaying a notable, dose-dependent responsiveness to GCs [183, 184].

As with CS, brain structure alterations are also described in patients with CAH. MRI studies have confirmed a significant reduction in whole brain volume and notably in the hippocampus, amygdala [185], thalamus, cerebellum, and brain stem [186], as well as alterations in areas closely associated with visuo-spatial and working memory (pre-frontal, parietal, and superior occipital cortex) [187]. Similarly, white matter [188–190] as well as gray matter [191] abnormalities in regions closely associated with cognitive functioning (hippocampus, hippocampal subiculum, and CA1 subregions) were described in patients with CAH [192–194]. Importantly, these alterations were not always related to GC dose, suggesting that over-treatment might not be the only factor involved in determining structural brain alterations. In fact, these alterations have also been recently linked with cortisol deficiency in patients with CAH [195]. The amygdala and hippocampus exhibit a high GR density [196] and are known to exert negative feedback on the HPA axis during the stress response [197]. Where HPA-axis function is dysregulated, feedback circuit disruption results in a lack of proliferation, cell death, and, consequently, smaller volumes in these areas [198], resulting in cognitive impairment.

It is likely that multiple factors, including prolonged exposure to androgen excess, cortisol deficiency, and GC-induced deterioration of brain regions, shape the cognitive impairment in patients with CAH [199, 200]. Other than pre-natal androgen excess, the role of pre-natal dexamethasone (DEX) therapy on cognitive function in CAH patients has been explored during the last decade. DEX treatment has been traditionally employed to prevent genital virilization in female fetuses at risk of CAH. The results are conflicting, and current guidelines refer to pre-natal DEX treatment as an *experimental therapy* [155], since the fetal risks from pre-natal DEX exposure outweigh the potential consequences of genital virilization [201]. Prenatal GC exposure can disrupt the HPA axis, enhancing the cortisol response to stress [202], with negative long-term consequences regarding mental health in childhood and adolescence [203]. Notably, DEX is not metabolized by 11β-HSD type 2 [103] and retains minimal (if not negligible) MC activity [204], which can be even more detrimental to the MR/GR activation ratio in MC-sensitive brain areas.

In utero*,* GC over-exposure increases the risk of affective, cognitive, and motor behavior impairment [205] and children treated prenatally with DEX have been reported to be less sociable, more emotional [206], and socially anxious [207] [206], albeit this was not confirmed in a recent meta-analysis [208] (probably due to the observational nature and the small sample size of the available studies). Similarly, the current evidence regarding cognitive function has yielded conflicting data. Several investigations reported no differences in general intelligence, long-term memory, and learning capabilities between children with CAH prenatally exposed to DEX versus those who were not. However, verbal working memory, self-perception for scholastic competence [207], and verbal intelligence [178] were found to be significantly reduced in the former group. Interestingly, a single study reported sex-specific long-term cognitive effects (slower mental processing) of pre-natal DEX in girls with CAH, but not boys [209]. The underpinning reasons are currently unknown.

In conclusion, patients with CAH have worse general intelligence and lower IQ compared to healthy controls, and cognitive impairment is often associated with disease severity. Adrenal crises, hyperandrogenaemia, cortisol deficiency, and GC dose regimens are all risk factors for cognitive decline in patients with CAH. This parallels with significant brain alterations seen at MRI, possibly secondary to abnormal brain development due to a mixture of *in-utero* hormonal imbalance and post-natal GC excess.

A summary of the current evidence for cognitive function in CS is shown in Table 1 and Supplemental Table 3.

## Exogenous glucocorticoids’ treatment and cognition

eGCs are among the most prescribed drugs in clinical practice, being a mainstay for the treatment of several autoimmune and inflammatory disorders [210]. In recent years, a notable increase in prescription rates [211, 212] and, subsequently, in eGC-associated side effects has been observed [212–215]. For instance, adverse psychiatric side effects (APSEs) frequently occur during eGC treatment, with a prevalence ranging from 3 to 60% of cases [216]. While psychosis, mania/hypomania, depression, and anxiety are the most common findings, long-lasting cognitive impairment is also described during eGC therapy, with a prevalence ranging from 0.4 [217] to 7% of cases [218].

Overall, prolonged eGC treatment is known to cause cognitive deficits [219–221], in a pattern of neurocognitive decline known as “*steroid dementia*” [217], typically characterized by deficits in declarative memory, mental processing speed, and concentration [222].

Interestingly, different eGCs might exert varying effects on cognitive functioning due to their specific impact on the MR/GR balance in the brain. Short- and intermediate-acting compounds (SIAGCs), such as cortisone, hydrocortisone, and prednisone (PRED), can activate both MR and GR, whereas longer-acting eGCs (LAGCs), like DEX and methylprednisolone (MP), preferentially bind to GR, suppressing endogenous cortisol production via negative feedback on the HPA and causing a decrease in MR occupation.

Indeed, the administration of hydrocortisone has been shown to partially improve memory deficits produced by the chronic administration of DEX [223]. Similarly, in a randomized-controlled trial conducted on 50 children treated with DEX (6 mg/m^2^/day for two 5-day courses) with acute lymphoblastic leukemia, the administration of 10 mg/m^2^/day of thrice-daily hydrocortisone in a circadian fashion (higher dosage given in the morning) markedly improved the DEX-induced APSEs [224]. These beneficial effects have been ascribed to the refill of unoccupied brain MRs, which is typically associated with SIAGCs, but not LAGCs, resulting in a restoration of the correct MR/GR activation balance. Intriguingly, the fludrocortisone-mediated activation of the MR has been demonstrated to improve memory in healthy individuals [126].

The direct comparison between the neurocognitive effects of DEX and PRED has yielded unexpected results: in pediatric patients with lymphoblastic leukemia, no differences were found in cognitive functioning [225], except for worse fluid reasoning, higher likelihood of enrolling in special education services [226] and word reading [227] in DEX-treated patients. Similarly, the available findings regarding the effects of SIAGCs on cognition are controversial. Mild, acute rises in cortisol, following the administration of low-to-moderate doses of hydrocortisone (< 25 mg), are known to enhance memory consolidation [228], and emotional and habit learning [229, 230] in healthy individuals. However, the short-term administration of SIAGCs has also been shown to adversely affect memory performance in both adults [217, 231] and children [232]. Several studies in healthy subjects have reported poor long-term memory retrieval [233, 234] (mainly impairments in autobiographical memory [235, 236] and recall performance of verbal material) after acute challenge with HC [118]. On the other hand, attention, vigilance [237], working memory, and verbal executive functions [234, 238] seem to be less affected by eGC administration. The discrepancy between these findings might reflect different designs, small sample sizes, and an overall heterogeneity of the studies.

In this regard, a meta-analysis with a total of 563 healthy volunteers identified two possible factors influencing the relationship between eGC administration and memory. First, administering eGCs before learning was not found to have a significant effect on memory, whereas when given prior to information recall, a significant mnemonic impairment was seen [118], mainly affecting the retrieval of declarative memory [233, 239].

Second, the time of the day in which eGCs are administered appears to be an important determinant of their cognitive effects. Specifically, administration of modest doses (i.e., hydrocortisone 20–40 mg) before cognitive testing in the afternoon results in mild memory enhancement [240], whereas a morning administration (a time in which GRs are already partially saturated by higher endogenous cortisol levels) is associated with memory impairment [118], likely due to the oversaturation of GRs [241].

The cognitive alterations in patients receiving eGCs resemble those observed in patients with CS (impaired memory and verbal learning) [45]. However, in the latter group, cognitive impairment is typically persistent, whereas the extent of the reversibility of mnemonic impairments in patients receiving eGCs is still a matter of debate. While several reports have documented complete cognitive recovery within weeks of eGCs’ discontinuation [242, 243], others observed a long-term persistence of mild cognitive decrement up to 1 year following suspension [217, 244], a divergence that may be attributable to the different duration and fluctuations in eGC exposure [45].

Another concern is whether eGCs’ administration might affect brain structure. In agreement with older evidence [245, 246], recent investigations have documented the incidence of hippocampal and cerebral atrophy following eGC treatment. Notably, brain atrophy has been reported to occur shortly after the acute administration of high-dose methylprednisolone (1 g daily for 3 consecutive days) in patients with multiple sclerosis, with reduced brain volume being observed up to 2 months following intravenous infusion [247, 248]. However, current evidence is still limited and often inconsistent [219, 245, 249], with early studies suggesting a potential correlation between the degree of brain atrophy with eGC dose [245]. Moreover, a clear recovery of brain architecture following treatment discontinuation has been documented in some, but not all patients [245, 250]. To date, no reliable predictors for structural brain recovery have been identified.

A correlation between treatment duration and degree of morphological alteration has been suggested in an MRI and proton magnetic resonance spectroscopy study on 17 patients on long-term prescription with PRED therapy (≥ 10 mg/day for ≥ 6 months). Compared to matched controls, patients with longer treatment durations had smaller HVs, atrophy of the amygdala, and decreased neuronal vitality [219]. In line with these findings, multiple functional imaging studies have confirmed that cortisone administration significantly reduces blood flow and glucose metabolism in memory-related brain regions (such as the posterior-medial temporal lobe [251] and hippocampus [252, 253]) and exert detrimental effects on the excitability, structure, and functionality of the pre-frontal cortex, with a related impairment of working memory [254–258].

It might be expected that longer exposure to eGC treatment would result in more severe cognitive impairment. However, the actual impact of treatment duration on memory function is unclear. Early work [220] evaluated cognition in patients receiving chronic PRED treatment (16.4 mg/day for more than one year). In accordance with similar findings in later studies [259, 260], patients receiving chronic eGC treatment performed significantly worse than controls on hippocampal-dependent memory tasks. However, in these studies, memory impairment was not influenced by the duration of treatment. A recent double-blind, placebo-controlled, crossover study compared cognitive function and brain MRI morphology in patients with rheumatoid arthritis, either treated with chronic PRED therapy (7.5 mg/day for 5 years) or without eGCs. No difference was found between the two groups regarding memory performance or HV. Interestingly, acute PRED challenge before cognitive testing resulted in impaired delayed verbal memory recall in both groups, suggesting that acute, rather than chronic, exposure is responsible for memory deficits in these contexts [239]. Collectively, further evidence is needed to clarify the role of treatment duration in the pathophysiology of eGCs-induced cognitive impairment.

It is interesting to speculate that aging might influence the brain’s susceptibility to eGC-induced cognitive decline. Indeed, greater memory decline [220, 237] following exposure to eGCs has been described in older, when compared to younger patients. However, this was not confirmed in another study [261] in which younger subjects showed impaired short-term working memory tests, suggesting that older individuals might be less responsive to acute GC challenge due to the physiological atrophy of the frontal lobe found in these subjects [261]. There is, therefore, currently no conclusive evidence as to the predictive role of age in eGC-induced cognitive decline.

In contrast, there is a much clearer association between eGCs doses and the development of cognitive decline and neuropsychiatric symptoms [45, 216]. APSEs rarely occur at PRED-equivalent doses of < 20 mg/day [262], but doses above 40 mg PRED-equivalent per day exhibit the highest risk of acute events [263] up to a proper “*steroid induced psychosis*”. This is a quite difficult condition to manage, and a multidisciplinary approach (including dedicated support from psychiatrists) is strongly suggested. The most effective treatment consists of a combined strategy: both GC dose reduction or discontinuation (if possible) and administration of antipsychotic medications are required for the *restitutio ad integrum* of negative or delusional and hallucinatory symptoms. Among the antipsychotic drugs, haloperidol and risperidone have exhibited the best efficacy profile [264, 265] with symptoms often resolving in few weeks [262]. Liver and kidney diseases and function need to be carefully assessed while examining the safety profile of the antipsychotic drug. Quetiapine, aripiprazole or olanzapine, as well as mood stabilizers, selective serotonin re-uptake inhibitors, can be considered as second-line treatments [264, 265].

Regarding cognitive functioning, the relation between SIAGC dose and memory typically follows an inverted *U*-shaped function [241, 266], facilitating delayed memory retrieval at a threshold dose of 20 mg/day of hydrocortisone (which mirrors the physiological cortisol increases during mild stress), with higher doses resulting in impaired cognitive function [241].

Early reports documented a striking dose–response correlation between PRED dose and APSEs in hospitalized patients [267]. High eGC dosing (> 160 mg of hydrocortisone equivalent) induces a reversible but significant decrease in declarative and autobiographical memory [237, 268, 269]. The same effects were confirmed in healthy volunteers receiving high-dose intravenous hydrocortisone (0.45 mg/kg/day) compared to those under lower dose (0.15 mg/kg/day) [270]. Similarly, the administration of 40 mg/day of oral hydrocortisone worsens delayed recall performances compared to 20 mg/day [241].

Interestingly, higher eGC doses have also been associated with earlier symptom appearance, with memory deficits occurring within 3–5 days from administration [268, 271] and quicker recovery following medication withdrawal [272].eGCs also affect emotional memory retention in a dose-dependent fashion: low-to-moderate eGCs doses (< 20 mg/day) have been shown to increase inhibition of negative emotional stimuli [273, 274], whereas higher doses (40 mg/day) facilitate the experience of negative emotions [275, 276].

It is important to note that, aside of the mentioned direct detrimental effects on cognitive function caused by eGC administration, tapering down longstanding supraphysiological dose when the underlying disease has subsided or is well controlled with alternative non-GC medications, often results in an enigmatic phenomenon referred to as the glucocorticoid withdrawal syndrome (GWS) [277, 278]. This syndrome represents a unique challenge for the endocrinologist and manifests with symptoms resembling AI, often including irritability, mood swings, and psychiatric symptoms (depression, anxiety, panic attacks, up to psychotic state) [85, 278, 279]. The mechanisms behind GWS probably depend on the developed dependence on supraphysiological GC concentrations but are still not entirely understood. The pathogenesis seems to be multifactorial and, among the proposed mechanisms, the downregulation of CRH and proopiomelanocortin, as well as the upregulation of mediators such as vasopressin, central noradrenergic, and dopaminergic systems, seem to mediate cognitive disruption [280]. Indeed, an intact CRH system in the brain is necessary for adequate mesolimbic dopaminergic function; its alteration contributes to inadequate stimulation of dopaminergic neurons terminating in the *nucleus accumbens*, fuelling anxiety and depression [280]. In this context, there are no studies defining possible predictors for GWS development. Albeit there are data demonstrating possible predictor for recovery of the HPA axis in patients treated with chronic eGC [281, 282], studies investigating the effects of different titrating protocols on GWS development have so far been inconclusive [283] and, as such, an individualized approach is needed. Cognitive therapy in parallel with antidepressants (fluoxetine, sertraline, and trazodone) can be helpful to target specific patient symptoms and improve mood [284].

In conclusion, eGCs influence cognitive function, most notably impairing declarative, hippocampus-dependent memory. Chronic administration generally results in memory impairment; however, short-acting formulations can exert variable cognitive effects, depending on dosage and administration timing. The contribution of age, treatment dose, and duration have yet to be clearly established. The hippocampus, amygdala, and pre-frontal cortex are particularly affected by eGC excess and display structural alterations, that appear to be only partially reversible following treatment discontinuation.

A summary of the current evidence for cognitive function during eGC is shown in Table 1*.*

## Conclusions

The HPA axis exerts important actions on the CNS in governing the physiological interactions between different brain areas involved in the cognition processes. GC fluctuation regulates a wide range of cognitive functions through a controlled interaction with GR and MR, guaranteed by substrate availability and receptor distribution. Any alterations in these complex processes can result in cognitive dysfunction. Due to the broad spectrum of unspecific symptoms complained by the patients, the recognition of cognitive deficit in patients with GC disorders is challenging, often delayed, or mistaken. Aside from neuropsychiatric symptoms, both hyper- and hypocortisolism, as well as exogenous steroid treatment, can all affect cognitive function (impacting mostly, but not only, memory), being the limbic system the more GC-sensitive brain area. A prompt recognition and treatment of underlying disease might be crucial to avoid permanent damage, albeit the resolution of hormonal imbalance does not guarantee complete recovery. Several authors are spending efforts to find possible pathogenetic factors and predictors for cognitive recovery after treatment. The contribution of absolute cortisol levels, the duration of exposure to altered cortisol concentrations or fluctuations, the balance between MR/GR activation, and the potential role of the androgen levels in CAH are all possible players involved in the damage. Sadly, to date, there are no accurate predictors for cognitive recovery following disease remission. Further studies are needed to find possible mechanisms involved to be targeted for future treatment strategies with the aim of a tailored precision-medicine approach.

### Supplementary Information

Below is the link to the electronic supplementary material.

Supplementary file1 (DOCX 47 KB)

Supplementary file2 (DOCX 31 KB)

Supplementary file3 (DOCX 34 KB)

## Data Availability

Data sharing not applicable to this article as no datasets were generated or analysed during the current study.
