# Supplementary material for: Glucocorticoids and cognitive function: a walkthrough in endogenous and exogenous alterations
Source: J Endocrinol Invest. 2023 Apr 14;46(10):1961–82. doi: 10.1007/s40618-023-02091-7 (PMC10514174; doi:10.1007/s40618-023-02091-7)
Supplement: Supplementary file 1 — Supplementary file1 (DOCX 47 KB) [file 40618_2023_2091_MOESM1_ESM.docx]

**Title:** *“Glucocorticoids and cognitive function: a walkthrough in endogenous and exogenous alterations”*

**Journal:**

Journal of Endocrinological Investigation

**Authors**:

Dario De Alcubierre^1^, Davide Ferrari^1^, Gianluca Mauro^2^, Andrea M Isidori^1^, Jeremy W Tomlinson^3^ and Riccardo Pofi^3^.

**Affiliations**

^1^ Department of Experimental Medicine, Sapienza University of Rome, Rome, Italy.

^2^ Department of Physiology and Pharmacology, Sapienza University of Rome, Rome, Italy.

^3^ Oxford Centre for Diabetes, Endocrinology and Metabolism, NIHR Oxford Biomedical Research Centre, University of Oxford, Churchill Hospital, Oxford, UK.

**Corresponding author**

Riccardo Pofi, Oxford Centre for Diabetes, Endocrinology and Metabolism, NIHR Oxford Biomedical Research Centre, University of Oxford, Churchill Hospital, Oxford, UK, riccardo.pofi@ocdem.ox.ac.uk.

**Supplemental Table 1.** Summary of the evidence concerning CS and cognition.

| **Authors** | **Year** | **Study design** | **Study population** | **Age** | **Assessments** | **Results** | | | | **Comments** |  |
| --- | --- | --- | --- | --- | --- | --- | --- | --- | --- | --- | --- |
| Starkman MN et al. [42] | 2001 | Case-control | **48 CD**  (F_37_M_11_)  **38 HS**  (F_30_M_8_) | **36.6Y**  (14.4)  **36.7Y**  (13.9) | General verbal intelligence, reasoning, arithmetics | ↓↓ | | | | Verbal impairment was higher than visual impairment. No association was found between depression score and cognitive performance. |  |
|  |  |  |  |  | Visuospatial abilities, attention and mental flexibility | ↓ | | | |  |  |
|  |  |  |  |  | Working memory | ↓ | | | |  |  |
|  |  |  |  |  | Verbal memory and learning | ↓↓ | | | |  |  |
|  |  |  |  |  | Visual memory and learning | ↓ | | | |  |  |
| Mauri M et al. [44] | 1993 | Case-control | **25 CD**  (F_17_M_8_)  **25 HS**  (F_17_M_8_) | **35.7Y**  (14.3) | Verbal memory (working/long term) | ↓ | | | | Cognitive performance did not correlate with anxiety/depression scores. 8 patients were rescreened after surgery and showed improvements in verbal memory, working memory and mental flexibility. |  |
|  |  |  |  |  | Visual memory (working/long term) | ↓ | | | |  |  |
|  |  |  |  |  | Mental flexibility | ↓ | | | |  |  |
|  |  |  |  |  | Executive functions (attention, concentration, processing speed, general reasoning, visuo-spatial-functioning) | = | | | |  |  |
| Michaud K et al. [50] | 2009 | Case control | **10 CS**  (F_8_M_2_)  **10 HS**  (F_8_M_2_)  **10 OC**  (F_8_M_2_) | **43.9Y**  (7.1)  **43.1Y** (8.6)  **59.7Y**  (6.9) |  | **CS** | **HS** | | **OC** | HS performed better than CS and OC in attention, visual processing, spatial memory and reasoning. CS and OC performed similarly on cognition tasks, suggesting an “aging-like” effect of chronic hypercortisolism on cognitive function. |  |
|  |  |  |  |  | Attention | ↓ | ↑ | | ↓ |  |  |
|  |  |  |  |  | Visual processing | ↓↓ | ↑ | | ↓ |  |  |
|  |  |  |  |  | Memory performance | ↓ | ↑ | | ↓ |  |  |
|  |  |  |  |  | Reasoning and concept formation | ↓ | ↑ | | ↓ |  |  |
|  |  |  |  |  | Verbal fluency | ↓ | ↑ | | ↓ |  |  |
|  |  |  |  |  | Depression (used as covariate) | ↑ | = | | = |  |  |
| Resmini E et al. [46] | 2012 | Case-control | **33 CS** (11 active and 22 cured)  (F_27_M_6_)  **34 HS**  (F_25_M_9_) | **44.8Y**  (11.8)  **41.4Y** (12.1) | Verbal memory, learning and recognition | ↓ | | | | No differences were found between active and cured CS.  Right HV at MRI was related to visual memory tasks while left HV was related to verbal memory tasks. |  |
|  |  |  |  |  | Visual memory and visuospatial abilities | ↓ | | | |  |  |
|  |  |  |  |  | Hippocampal volume | = | | | |  |  |
|  |  |  |  |  | Cortical and total GM | ↓ | | | |  |  |
| Forget H et al [48] | 2016 | Case-control and longitudinal pre/post-treatment | **18 CS**  (F_15_M_3_)  **18 HS**  (F_15_M_3_) | **39.6Y** (9.8)  **41.7Y** (9.5) |  | **BASE** | | **F-U** | |  |  |
|  |  |  |  |  | Attention, processing speed | ↓ | | =/↑ | | BASE = baseline evaluation (compared to HS).  F-U: post-treatment follow-up evaluation (up to 36 months).  Verbal memory was better preserved than nonverbal memory. |  |
|  |  |  |  |  | Visual processing | =/↓ | | ↑ | |  |  |
|  |  |  |  |  | Learning and memory | = | | ↑ | |  |  |
|  |  |  |  |  | Executive functions (processing speed, general reasoning) | ↓ | | = | |  |  |
|  |  |  |  |  | Verbal intelligence | ↓ | | ↑ | |  |  |
|  |  |  |  |  | Global intelligence | ↓ | | ↑ | |  |  |
| Bourdeau I et al. [58] | 2002 | Case-control and longitudinal pre/post-treatment | **21 CD**  (F_13_M_8_)  **17 ACS**  (F_16_M_1_)  **18 OST**  (F_14_M_4_)  **20 NST**  (F_17_M_3_) | **37.3Y** (11.1)  **45.4Y** (12.0)  **35.4Y** (8.4)  **37.3Y** (12.8) | Third ventricle diameter | Reduced in CD and ACS compared to NST | | | | 22 CS were re-evaluated after treatment (mean follow-up duration 15.5 months, 7-24). Within this cohort, the three MRI parameters of brain atrophy improved after correction of hypercortisolism. |  |
|  |  |  |  |  | Bicaudate diameter |  |  |  |  |  |  |
|  |  |  |  |  | Subjective evaluation scale of atrophy |  |  |  |  |  |  |
| Santos A et al. [60] | 2014 | Case-control | **15 active CS**  (F_13_M_2_)  **21 cured CS**  (F_17_M_4_)  **36 HS**  (F_30_M_6_) | **44.2Y** (9.3)  **41.9Y** (10.4)  **42.7Y** (9.9) | Verbal memory (working and long term), fluency, vocabulary, learning | = | | | | Active CS showed smaller bilateral cerebellar cortex at MRI and impaired visual memory compared to HS. No differences were found between cured CS and HS. |  |
|  |  |  |  |  | Visuospatial abilities | = | | | |  |  |
|  |  |  |  |  | Visual memory (working and long-term) | ↓ | | | |  |  |
|  |  |  |  |  | Executive functions (Attention, processing speed, flexibility, coordination and motor speed) | = | | | |  |  |
|  |  |  |  |  | Cerebellar cortex | ↓ | | | |  |  |
|  |  |  |  |  | Cerebellar WM and total volume | = | | | |  |  |
| Jiang H et al. [61] | 2017 | Case-Control, treatment-effect | **20 active CD**  (F_17_M_3_)  **14 STR-CD**  (F_9_M_5_)  **34 HS**  (F_26_M_8_) | **40.4Y** (11.3)**39.9Y** (17.3)  **44.5Y** (13.0) | Grey matter volume (MRI) | Compared to HS, GM volumes in medial frontal gyrus and cerebellum were lower in active CD but not in STR-CD. In bilateral caudate, STR-CD showed higher GM volumes than HS and active CD. In STR-CD, remission time correlated with GM values. | | | | | |
| Tirosh A et al. [63] | 2020 | Case-Control | **8 active CS**  (F_6_M_2_)  **21 cured CS**  (F_12_M_9_)  **8 HS**  (F_3_M_5_) | **15.5Y**  (10.8)  **21.0Y** (10.5)  **14.5Y** (25.5) | Brain structures volume, intensity and thickness (MRI) | 24h UFC positively correlated with lateral ventricles volumes and negatively with the intensity of subcortical GM, corpus callosum and cerebral WM and with the volume of several other CNS structures. LNPC negatively correlated with WM intensity of FLs and PLs.  Compared to cured CS, active CS showed increased WM volume and decreased cortex thickness and WM intensity mainly in FLs and PLs.  Compared to HS, cured CS showed lower subcortical GM volume, increased cortical thickness and decreased WM volume in multiple anatomic sites. | | | | | |
| Cui M et al. [65] | 2021 | Case-Control | **58 CD**  (F_53_M_5_)  **54 HS**  (F_51_M_3_) | **37.9Y**  (10.7)  **34.6Y**  (10.7) | WM Fractional Anisotropy (FA) | ↓ | | | | At MRI-DTI, CD showed a peculiar WM microstructural pattern (increase in MD and RD, FA decrease and partial AD increase) in different CNS tracts. Compromised WM microstructure correlated with ACTH and cortisol concentration and cognitive decline. |  |
|  |  |  |  |  | WM Mean Diffusivity (MD) | ↑ | | | |  |  |
|  |  |  |  |  | WM Radial Diffusivity (RD) | ↑ | | | |  |  |
|  |  |  |  |  | WM Axial Diffusivity (AD) | =/↑ | | | |  |  |
|  |  |  |  |  | General Cognition (MMSE/MoCA) | ↓ | | | |  |  |
|  |  |  |  |  | Depressive symptoms | ↓ | | | |  |  |
| Pupier E et al. [77] | 2022 | Case-Control | **25 cured CD**  (F_19_M_6_)  **25 HS**  (F_19_M_6_) | **44.5Y** (2.4)  **44.3Y** (2.3) | Verbal memory, learning and recognition | = | | | | Mean remission duration was 102.7 (19.3) months.  Despite a worse reported quality of life, cured CD did not show any difference in cognitive function compared to HS. |  |
|  |  |  |  |  | Verbal fluency | = | | | |  |  |
|  |  |  |  |  | Visual memory and visuospatial functioning | = | | | |  |  |
| Bauduin S et al. [86] | 2020 | Case-Control | **25 cured CS**  (F_21_M_4_)  **25 HS**  (F_21_M_4_) | **45.0Y**  (8.0)  **47.0Y**  (7.0) | Self-reported cognitive function | ↓ | | | | At MRI, compared to HS, long-term remitted CD (0.8-29.3 years) showed smaller cortical thickness of several limbic areas, key regions for emotional and cognitive processing. Left caudal ACC thickness correlated with disease duration and anxiety symptoms. |  |
|  |  |  |  |  | Cortical thickness | ↓ | | | |  |  |
|  |  |  |  |  | Surface area of cortex | =/↑ | | | |  |  |
| Pires P et al.  64, 66] | 2015 - 2017 | Case-Control | **8 active CS**  (F_7_M_1_)  **7 remitted CS**  (F_6_M_1_)  **20 cured CS**  (F_16_M_4_)  **35 HS**  (F_29_M_6_) | **41.9Y**  (8.7)  **47.1Y**  (10.0)  **41.4Y**  (10.0)  **42.3Y**  (10.4) | WM Fractional Anisotropy (FA) | ↓ | | | | Compared to HS, CS showed widespread WM alterations at MRI-DTI indicative of loss of integrity and demyelination even after correcting for cardiovascular risk factors. No differences were found in WM alterations between cured, remitted and active CS, suggesting that the damage is not (at least completely) reversible. Depression (but not anxiety) and processing speed were related to WM parameters. |  |
|  |  |  |  |  | WM Mean Diffusivity (MD) | ↑ | | | |  |  |
|  |  |  |  |  | WM Radial Diffusivity (RD) | ↑ | | | |  |  |
|  |  |  |  |  | WM Axial Diffusivity (AD) | ↑ | | | |  |  |
|  |  |  |  |  | Executive functions (processing speed) | = | | | |  |  |
|  |  |  |  |  | Depression and anxiety | ↑  (worse) | | | |  |  |
| Forget H et al.  [47, 78] | 2000  -  2002 | Case-Control and longitudinal pre/post-surgery | **19 CS (11 CD, 8 ACS)**  (F_18_M_1_)  **19 HS**  (F_18_M_1_)  13 CS (9 CD 4 ACS) were evaluated at least 1 year after surgery | **46.8Y**  (11.0)  **47.1Y**  (11.7) |  | **BASE** | | **F-U** | | BASE = baseline evaluation (compared to HS); F-U= follow-up evaluation after successful surgical treatment (compared to baseline). Except for a slight improvement in visual organization, normalization of cortisol level did not ameliorate cognitive decline. |  |
|  |  |  |  |  | Verbal intelligence (IQ) and fluency | ↓ | | = | |  |  |
|  |  |  |  |  | Visuospatial intelligence (IQ) | ↓ | | = | |  |  |
|  |  |  |  |  | Global intelligence (IQ) | ↓ | | = | |  |  |
|  |  |  |  |  | Executive functioning (visuospatial abilities, selective and global attention, processing speed, conceptualization, reasoning) | ↓ | | = | |  |  |
|  |  |  |  |  | Working memory / verbal memory | =/↓ | | = | |  |  |
|  |  |  |  |  | Visual memory | ↓ | | = | |  |  |
|  |  |  |  |  | Visual recognition | = | | = | |  |  |
| Hook JN et al. [75] | 2007 | Longitudinal pre/post-treatment study | **27 CD**  (F_23_M_4_) | **38.7Y** (13.2) | Verbal learning/memory | ↑ | | | | The improvement in verbal memory was related to hippocampal formation volume.  Age, independently from disease duration, was found to significantly affect the speed of recovery of cognitive function. |  |
|  |  |  |  |  | Working memory and attention | = | | | |  |  |
|  |  |  |  |  | Verbal fluency | ↑ | | | |  |  |
|  |  |  |  |  | Depression | = | | | |  |  |
|  |  |  |  |  | Hippocampal formation volume | ↑ | | | |  |  |
|  |  |  |  |  | Caudate head volume | = | | | |  |  |
| Ragnarsson O et al. [79] | 2012 | Case-Control | **55 treated CS** (43 CD, 12 ACS)  (F_50_M_5_)  **55 HS**  (F_50_M_5_) | **52.9Y**  (14.1)  **53.0Y**  (14.3) | Working memory | ↓ | | | | Median duration of remission was 13 (5-19) years.  No differences were found between ACS and CD. Concomitant affective disorders and chronic fatigue were unrelated to cognitive performance. |  |
|  |  |  |  |  | Visuospatial functioning (orienting and alerting) | ↓ | | | |  |  |
|  |  |  |  |  | Auditory attention | ↓ | | | |  |  |
|  |  |  |  |  | Processing speed | = | | | |  |  |
|  |  |  |  |  | Verbal fluency and reading speed | ↓ | | | |  |  |
| Papakokkinou E et al. [80] | 2015 | Case-Control | **51 treated CS** (39 CD, 12 ACS)  (F_47_M_4_)  **51 HS**  (F_47_M_4_) | **52.5Y**  (14.6)  **53.6Y**  (13.9) | Mental fatigue (exhaustion and long recovery time following mentally strenuous tasks) | ↑  (worse) | | | | Median duration of remission was 12 (4-18) years.  CD and ACS showed no differences in attention tasks. Radiotherapy was related to worse performance in 1 attention task. Mental fatigue was related to the attention performance. |  |
|  |  |  |  |  | Executive functioning (attention) | ↓ | | | |  |  |
| Hou B et al. [87] | 2020 | Case-Control and longitudinal pre/post-surgery | **50 CD**  (F_10_M_50_)  **36 HS**  (F_25_M_11_) | **31.7Y**  (10.0)  **35.8Y**  (10.6) |  | **BASE** | | **F-U** | | BASE = baseline evaluation (compared to HS)  F-U = 3 months follow-up after surgery (in 46/50 patients that remitted) compared to baseline.  MRI scans were analyzed with artificial intelligence. Age, disease duration showed no significant correlation with brain volumes. |  |
|  |  |  |  |  | Total GM volume | ↓ | | ↑ | |  |  |
|  |  |  |  |  | Total WM volume | = | | = | |  |  |
|  |  |  |  |  | Frontal lobes volume | ↓ | | = | |  |  |
|  |  |  |  |  | Temporal lobes volume | ↓ | | = | |  |  |
|  |  |  |  |  | Occipital and Parietal lobes volume | ↓ | | ↑ | |  |  |
|  |  |  |  |  | Hippocampus and amygdala volume | = | | = | |  |  |
|  |  |  |  |  | Insula, cingulate, ventricles volume | ↓ | | ↑ | |  |  |
| Starkman MN et al. [82] | 1999 | Longitudinal pre/post-surgery | **22 CS**  (F_17_M_5_) | **38.7Y**  (14.8) | Hippocampal formation volume (HFV) | ↑ | | | | Mean follow-up duration was 16 (9.3) months. At MRI, HFV increase after surgery was greater than that of the caudate head (comparison structure). The reduction in UFC was positively related to the percent increase in HFV. |  |
|  |  |  |  |  | Caudate head volume | =/↑ | | | |  |  |
| Crespo I et al. [84] | 2014 | Case-Control | **27 cured CS** (24 CD 3 ACS)  (F_22_M_5_)  **8 medically treated CS** (4CD, 3ACS, 1 ECS)  (F_8_M_0_)  **35 HS**  (F_30_M_5_) | **44.5Y**  (10.0)  **41.4Y** (12.3)  **44.4Y**  (9.5) | Decision making, strategy and learning | ↓ | | | | Globally, CS showed worse decision making and cortical thickness at MRI in several brain areas. There were no differences between cured and medically treated CS, indicating that brain changes and cognitive impairment are not completely reversible after remission. |  |
|  |  |  |  |  | Cortical thickness | ↓ | | | |  |  |

ACC = Anterior Cingulate Cortex, ACS = Adrenal Cushing’s Syndrome, CD = Cushing’s Disease, CNS = Central Nervous System, CS = Cushing’s Syndrome, DTI = Diffusion Tensor Imaging, ECS = Ectopic Cushing’s Syndrome, FLs = Frontal Lobes, GM = Grey Matter, HS = Healthy subjects, HV = Hippocampal Volume, IQ = Intelligence Quotient, LNPC = Late Night Plasma Cortisol, MMSE = Mini-Mental State Examination, MoCA = Montreal Cognitive Assessment, MRI = Magnetic Resonance Imaging, NST = No Sellar Tumors, OC = Older Controls, OST = Other (non-ACTH-secreting) sellar tumors, PLs = Parietal Lobes, UFC = Urinary Free Cortisol, STR-CD = Short-Term Remission Cushing’s Disease, WM = White Matter,
