# Supplementary material for: Glucocorticoids and cognitive function: a walkthrough in endogenous and exogenous alterations
Source: J Endocrinol Invest. 2023 Apr 14;46(10):1961–82. doi: 10.1007/s40618-023-02091-7 (PMC10514174; doi:10.1007/s40618-023-02091-7)
Supplement: Supplementary file 2 — Supplementary file2 (DOCX 31 KB) [file 40618_2023_2091_MOESM2_ESM.docx]

**Title:** *“Glucocorticoids and cognitive function: a walkthrough in endogenous and exogenous alterations”*

**Journal:**

Journal of Endocrinological Investigation

**Authors**:

Dario De Alcubierre^1^, Davide Ferrari^1^, Gianluca Mauro^2^, Andrea M Isidori^1^, Jeremy W Tomlinson^3^ and Riccardo Pofi^3^.

**Affiliations**

^1^ Department of Experimental Medicine, Sapienza University of Rome, Rome, Italy.

^2^ Department of Physiology and Pharmacology, Sapienza University of Rome, Rome, Italy.

^3^ Oxford Centre for Diabetes, Endocrinology and Metabolism, NIHR Oxford Biomedical Research Centre, University of Oxford, Churchill Hospital, Oxford, UK.

**Corresponding author**

Riccardo Pofi, Oxford Centre for Diabetes, Endocrinology and Metabolism, NIHR Oxford Biomedical Research Centre, University of Oxford, Churchill Hospital, Oxford, UK, riccardo.pofi@ocdem.ox.ac.uk.

**Supplemental Table 2.** Summary of the evidence concerning AI and cognition.

| **Authors** | **Year** | **Study design** | **Study population** | **Mean Age** (SD) | **Assessments** | **Results** | | | | | | **Comments** |
| --- | --- | --- | --- | --- | --- | --- | --- | --- | --- | --- | --- | --- |
| Henry M et al [96] | 2014 | Case-control | **27 PAI**  (M_7_F_20_)  **27 HS**  (M_7_F_20_) | **48.7Y**  (15.4)  **49.0Y**  (15.1) | Verbal learning | = | | | | | | Phone interview. |
|  |  |  |  |  | Verbal memory | ↓ | | | | | |  |
|  |  |  |  |  | Working memory | = | | | | | |  |
|  |  |  |  |  | Mental flexibility / task switching / reasoning | = | | | | | |  |
|  |  |  |  |  | Processing speed | ↓ | | | | | |  |
| Schultebraucks K et al. [99] | 2015 | Case-control | **30 PAI**  (M_9_F_21_)  **30 HS**  (M_9_F_21_) | **52.4Y**  (14.4)  **52.0Y**  (13.8) | Verbal learning | ↓ | | | | | | AI patients showed more depressive symptoms. |
|  |  |  |  |  | Verbal memory | = | | | | | |  |
|  |  |  |  |  | Visuo-spatial functioning | = | | | | | |  |
|  |  |  |  |  | Working memory | = | | | | | |  |
|  |  |  |  |  | Selective and general attention | = | | | | | |  |
| Klement J et al. [101] | 2009 | Case-control | **8 PAI**  (M_2_F_6_)  **8 HS**  (M_2_F_6_) | **52.6Y**  (3.2)  **52.1Y**  (3.9) | Verbal learning / memory | = | | | | | | Intravenous glucose infusion did not modify neurocognitive parameters. |
|  |  |  |  |  | Selective attention | ↓ | | | | | |  |
| Tiemensma J et al. [100] | 2016 | Case-control | **31 PAI**  (M_11_F_20_)  **31 HS**  (M_11_F_20_) | **49.0Y**  (12.4)  **45.0Y**  (12.0) | Logical memory | ↓ | | | | | | Postponement of HC dose did not change cognitive performance. |
|  |  |  |  |  | Verbal learning / memory | ↓ | | | | | |  |
|  |  |  |  |  | Visual learning / memory | ↓ | | | | | |  |
|  |  |  |  |  | Mental flexibility | ↓ | | | | | |  |
|  |  |  |  |  | Visuo-spatial functioning | ↓ | | | | | |  |
|  |  |  |  |  | Attention / Concentration | ↑ | | | | | |  |
|  |  |  |  |  | Mental flexibility / processing speed | = | | | | | |  |
|  |  |  |  |  | Sustained and selective attention | = | | | | | |  |
| Tytherleigh MY et al. [98] | 2004 | Pilot study | **9 PAI**  (gender not specified) | **37.9Y** (9.5) |  | **D** | **F** | | | **D+F** | | **D** = only DEX treatment 1 mg/day (only GRs); **F**= only Fludrocortisone treatment 0.2 mg/day (only MRs); **D+F** = Combined fludrocortisone 0.2 mg/day and DEX 1mg/day treatment (GRs/MRs) |
|  |  |  |  |  | Verbal learning / memory | ↓ | ↓ | | | ↑ | |  |
|  |  |  |  |  | Verbal learning / attention | = | = | | | = | |  |
|  |  |  |  |  | Working memory | ↓ | ↓ | | | ↑ | |  |
|  |  |  |  |  | Semantic memory / processing speed / mental flexibility | = | = | | | = | |  |
| Schultebraucks K et al. [109] | 2016 | Cohort study | **30 PAI**  (M_9_F_21_) | **52.4Y**  (14.4) |  | **NF** | | | **F** | | | Prior (NF) vs after (F) daily Fludrocortisone dose (Low MR occupation vs High MR occupation). |
|  |  |  |  |  | Verbal learning | ↓ | | | ↑ | | |  |
|  |  |  |  |  | Visuo-spatial functioning | = | | | = | | |  |
|  |  |  |  |  | Working memory | = | | | = | | |  |
|  |  |  |  |  | Selective and general attention | =/↓ | | | =/↑ | | |  |
| Henry M et al. [132] | 2017 | Case-control | **10 PAI**  (M_2_F_8_)  **10 HS**  (M_2_F_8_) | **42.0Y**  (10.1)  **40.3Y**  (11.6) | Verbal learning / memory | ↑ | | | | | | Sleep improved verbal memory with retention of more words in HS but not in PAI patients. |
|  |  |  |  |  | Procedural memory | = | | | | | |  |
| Henry M et al. [125] | 2015 | Case-control | **60 PAI**  (M_14_F_46_)  **60 HS**  (M_14_F_46_) | **50.6Y**  (14.4)  **49.9Y**  (14.7) | Clumsiness | ↓ | | | | | | Latent variable model showed that having poorer sleep would result in worse cognition. |
|  |  |  |  |  | Intention forgotten | ↓ | | | | | |  |
|  |  |  |  |  | Retrieval | ↓ | | | | | |  |
|  |  |  |  |  | Sleep quality | ↓ | | | | | |  |
| Harbeck et al. [102] | 2009 | Pilot study | **5 PAI 9 SAI**  (M_3_F_11_) | **29-70Y** |  | **NHC** | | | **HC** | | | Night infusions of Hydrocortisone (NHC) to mimick physiological night cortisol peak did not improve cognition.  Higher cortisol levels were associated to lower cognitive performance. |
|  |  |  |  |  | Intellectual functioning | = | | | = | | |  |
|  |  |  |  |  | Mental flexibility | = | | | ↑ | | |  |
|  |  |  |  |  | Working memory | = | | | = | | |  |
|  |  |  |  |  | Vigilance / attention / concentration | = | | | = | | |  |
|  |  |  |  |  | Visuo-spatial functioning | = | | | = | | |  |
| Krekeler et al [130] | 2021 | Prospective cohort study | **DR-HC**  10 PAI 8 SAI  (M_2_F_16_)  **Conv. HC**  10 PAI 8 SAI  (M_2_F_16_)  **9 HS** | **49.9Y**  (12.4)  **50.2Y**  (12.0) |  | **DRHC** | | **HC** | | | **HS** | DR-HC group (especially PAI patients) scored better than HC group in the field of intellectual abilities and visuo-spatial functioning. |
|  |  |  |  |  | Fluid intelligence / mental flexibility | = | | = | | | = |  |
|  |  |  |  |  | Working memory | = | | = | | | = |  |
|  |  |  |  |  | Visuo-spatial functioning | = | | = | | | = |  |
|  |  |  |  |  | Focused attention / concentration / vigilance | = | | = | | | = |  |
|  |  |  |  |  | Word knowledge | ↓ | | ↓ | | | ↑ |  |
|  |  |  |  |  | Attention (alertness) | ↓ | | ↓ | | | ↑ |  |
|  |  |  |  |  | Attention (reaction exchange) | ↑ | | ↓ | | | ↓ |  |
| Blacha AK et al. [114] | 2021 | Case-Control | **21 PAI 19 SAI**  (M_12_F_28_)  **20 HS**  (M_9_F_11_) | **52.7Y**  **51.6Y** | Fluid intelligence / intellectual functioning / word knowledge | = | | | | | | No differences between PAI and SAI were found except for a trend for worse vigilance.  Higher GC dosage was associated with worse performance in attention, visuospatial functioning and processing speed (executive functions). |
|  |  |  |  |  | Mental flexibility /concentration | = | | | | | |  |
|  |  |  |  |  | Visuo-spatial functioning | = | | | | | |  |
|  |  |  |  |  | Concentration / focused attention | = | | | | | |  |
|  |  |  |  |  | Attention (vigilance, alertness, reaction exchange) | ↓ | | | | | |  |
| Van't Westeinde A et al. [131] | 2022 | Case-control | **67 PAI**  (M_28_F_39_)  **80 HS**  (M_37_F_43_) | **32.3Y**  (6.7)  **29.2Y** (7.4) | Non-verbal intelligence | = | | | | | | PAI scores were within average range compared to normative data, HS scores were above population norms.  Female PAI reported more issues with some self-organization and emotional regulation than HS. |
|  |  |  |  |  | Verbal intelligence | =/↓ | | | | | |  |
|  |  |  |  |  | Verbal learning | = | | | | | |  |
|  |  |  |  |  | Working memory | =/↓ | | | | | |  |
|  |  |  |  |  | Executive functions (selective attention, processing speed) | = | | | | | |  |

Conv. HC = Conventional short-acting Hydrocortisone, DR-HC = Dual-Release Hydrocortisone, GRs = Glucocorticoid Receptors, MR = Mineracorticoid Receptors, HC = Hydrocortisone, HS = healthy subjects, PAI = Primary Adrenal Insufficiency, SAI = Secondary Adrenal Insufficiency
