# Supplementary material for: Glucocorticoids and cognitive function: a walkthrough in endogenous and exogenous alterations
Source: J Endocrinol Invest. 2023 Apr 14;46(10):1961–82. doi: 10.1007/s40618-023-02091-7 (PMC10514174; doi:10.1007/s40618-023-02091-7)
Supplement: Supplementary file 3 — Supplementary file3 (DOCX 34 KB) [file 40618_2023_2091_MOESM3_ESM.docx]

**Title:** *“Glucocorticoids and cognitive function: a walkthrough in endogenous and exogenous alterations”*

**Journal:**

Journal of Endocrinological Investigation

**Authors**:

Dario De Alcubierre^1^, Davide Ferrari^1^, Gianluca Mauro^2^, Andrea M Isidori^1^, Jeremy W Tomlinson^3^ and Riccardo Pofi^3^.

**Affiliations**

^1^ Department of Experimental Medicine, Sapienza University of Rome, Rome, Italy.

^2^ Department of Physiology and Pharmacology, Sapienza University of Rome, Rome, Italy.

^3^ Oxford Centre for Diabetes, Endocrinology and Metabolism, NIHR Oxford Biomedical Research Centre, University of Oxford, Churchill Hospital, Oxford, UK.

**Corresponding author**

Riccardo Pofi, Oxford Centre for Diabetes, Endocrinology and Metabolism, NIHR Oxford Biomedical Research Centre, University of Oxford, Churchill Hospital, Oxford, UK, riccardo.pofi@ocdem.ox.ac.uk.

**Supplemental Table 3**. CAH studies assessing cognitive and brain function.

| **Authors** | **Year** | **Study design** | **Study population** | **Mean Age** (SD) | **Assessments** | **Results** | **Comments** |
| --- | --- | --- | --- | --- | --- | --- | --- |
| Nass R et al. [148] | 1991 | Case-control with family members | **38 CAH**  20^SW^ 18^SV^  (F_18_M_20_)  **58 HS^S^**  (M_31_F_27_)  **52 HS^P^**  (F_28_M_24_) | **12.0Y**  (6.1)  **14.8Y**  (5.8) | Verbal intelligence | = | Verbal-Performance IQ discrepancy was larger in female CAH (especially in SV) than healthy female siblings and was in male range. SV were more likely to be learning disabled than SW and SW had a lower IQ than SV. |
|  |  |  |  |  | Nonverbal / visuospatial functioning | = |  |
|  |  |  |  |  | General intelligence | = |  |
| Browne WV et al. [139] | 2015 | Case-control with family members | **57 CAH**  (F_31_M_26_)  **50 HS^SC^**  (F_30_M_20_) | **8.6Y**  (1.3)  **9.1Y**  (1.7) | Working Memory | ↓ | No sex differences were observed. |
|  |  |  |  |  | Verbal fluency / intelligence | = |  |
| Hamed SA et al. [146] | 2018 | Case-control | **36 SW-CAH**  (F_24_M_12_)  **36 HS**  (F_25_M_11_) | **15.6Y**  (2.3)  **14.3Y**  (2.8) | Verbal intelligence (IQ) | = | No sex differences or type of GC treatment differences were observed. Poorly controlled patients showed lower IQ. GC dose and number of hyponatriemic episodes were indipendent risk factors of lower IQ. |
|  |  |  |  |  | Nonverbal / visuospatial intelligence (IQ) | ↓ |  |
|  |  |  |  |  | General intelligence (IQ) | ↓ |  |
|  |  |  |  |  | Short-term memory | ↓ |  |
|  |  |  |  |  | Verbal, visual and quantitative Reasoning | ↓ |  |
| Amr NH et al. [144] | 2019 | Case-control | **30 CAH**  (F_19_M_11_)  **20 HS**  (F_8_M_12_) | **10.2Y**  (3.2)  **11.0Y**  (2.7) | Verbal and general intelligence | ↓ | No differences were found after stratifying for daily GC dose.  Cumulative GC dose negatively correlated with visual perception/memory scores. |
|  |  |  |  |  | Nonverbal / visuospatial functioning / visual memory | ↓ |  |
|  |  |  |  |  | Executive functioning (planning, mental flexibility, task shifting, sustained attention) | ↓ |  |
| Messina V et al. [161] | 2020 | Case-control | **43 CAH** (11^PN-DEX^)  30^SW^ 12^SV^ 1^NCCAH^  (F_23_M_20_)  **52 HS**  (F_27_M_25_) | **7-17Y**  **7**-**17Y** | Verbal fluency, learning and memory | = | SW phenotype had lower scores than SV in visuospatial working memory. Different genotypes did not show differences in cognitive performance.  PN-DEX girls showed lower verbal intelligence while no differences were found in PN-DEX boys. |
|  |  |  |  |  | Nonverbal / visuospatial functioning and memory | = |  |
|  |  |  |  |  | Executive functions (working memory, selective attention, information processing) | = |  |
|  |  |  |  |  | General intelligence | = |  |
| Herting MM et al. [177] | 2020 | Case-control | **27 CAH**  (F_16_M_11_)  **35 HS**  (F_20_M_15_) | **12.6Y**  (3.4)  **13.0Y**  (2.8) | Intracranial volume | ↓ |  |
|  |  |  |  |  | Cerebrospinal fluid | ↑ |  |
|  |  |  |  |  | Bilateral SMF and CMF, left LOF | ↓ |  |
|  |  |  |  |  | Left hippocampus (TML)   - Bilateral subiculum and CA1 regions of hippocampus - Lateral nucleus of the amygdala | ↓ |  |
|  |  |  |  |  | Other brain areas | = |  |
| Mueller SC et al. [159] | 2008 | Case-control | **54 CAH**  25^SW^13^SV^16^NCCAH^  (F_31_M_23_)  **55 HS**  (F_22_M_33_) | **17.5Y**  **19.0Y** | Spatial cognition | SW females performed similarly to HS and CAH males.  Advanced bone age was correlated with improved performance. | |
| Karlsson L et al. [141] | 2017 | Case-control | **55 CAH** (9^PN-DEX^)  (F_30_M_25_)  **58 HS**  (F_31_M_27_) | **24.3Y**  (6.0)  **20.7Y**  (3.1) | Verbal and nonverbal fluency / intelligence | = | CAH men performed worse than HS men in terms of fluency/intelligence.  Null genotype, but not SW phenotype, was associated to worse fluency/reasoning.  PN-DEX women showed worse scores in most of the cognitive measures. |
|  |  |  |  |  | Executive functioning (working memory, visuospatial functioning, selective attention) | ↓ |  |
|  |  |  |  |  | Reasoning | = |  |
|  |  |  |  |  | Learning and memory | = |  |
| Johannsen TH et al. [147] | 2006 | Case-control | **35 CAH** **women**  **35 HS women** | **17-51Y**  **17-51Y** | Verbal intelligence | ↓ | SW had lower total and verbal IQs than SV and lower performance IQ than NCCAH. SV and NCCAH did not show any difference. |
|  |  |  |  |  | Nonverbal / visuospatial functioning | ↓ |  |
|  |  |  |  |  | General intelligence | ↓ |  |
| Van’t Westeinde A et al. [170] | 2020 | Case-control | **45** CAH (8^PN-DEX^)  (F_23_M_22_)  **43 HS**  (F_26_M_17_) | **23.1Y**  (4.9) | Verbal and nonverbal fluency / intelligence | = | PN-DEX affected brain structures in parietal and occipital cortex.  CAH patients showed alterations in prefrontal, parietal and superior occipital cortex, that encode for visuospatial working memory  After correcting for brain volume, the WM microstructure alterations were not confirmed. |
|  |  |  |  |  | Executive functioning (working memory, reasoning, selective attention) | = |  |
|  |  |  |  |  | Visuospatial working memory | ↓ |  |
|  |  |  |  |  | Learning and memory | = |  |
|  |  |  |  |  | Whole brain volume | ↓ |  |
|  |  |  |  |  | - Fractional anisotropy - Mean diffusivity - Radial diffusivity | =/↓  =/↓  =/↓ |  |
| Merke DP et al. [168] | 2003 | Case-control | **27 CAH**  13^SW^14^SV^  (F_11_M_16_)  **47 HS**  (F_13_M_34_) | **9.7Y**  (3.5)  **10.6Y**  (3.7) | Total brain and ventricles volume | = | Both CAH and HS children had above average IQ scores. CAH females showed a trend for a decreased cerebral volume. Females with CAH did not have brains with male-specific characteristics. |
|  |  |  |  |  | Temporal lobes and hippocampus volume | = |  |
|  |  |  |  |  | Amygdala volume | ↓ |  |
|  |  |  |  |  | General intelligence (IQ) | = |  |
| Mnif MF et al.  [176] | 2013 | Cross-sectional study | **26 CAH**  10^SW^ 8^SV^ 9^NCCAH^  (F_15_M_11_) | **27.4Y**  (8.2) | Neurologic signs | 15.5% | CAH patients showed increased frequency of WM abnormalities and temporal lobe structures dysgenesis. |
|  |  |  |  |  | WM hyperintensity | 30.7% |  |
|  |  |  |  |  | Temporal lobe/hippocampus dysgenesis | 11.5% |  |
|  |  |  |  |  | Other MRI signs | 11.5% |  |

17OHP = 17α-hydroxyprogesterone, CAH = Congenital Adrenal Hyperplasia, CMF = Caudal Middle Frontal cortex, GC = Glucocorticoids, HS = Healthy Subjects, HS^S^ = Healthy Siblings (brothers and sisters), HS^SC^ = Healthy Siblings and Cousins, HS^P^ = Healthy Parents (fathers and mothers), IQ = Intelligence Quotient, LOF = Lateral Orbito-Frontal cortex, MRI = Magnetic Resonance Imaging, NCCAH = Non Classical CAH, PN-DEX = CAH patients prenatally treated with Dexametasone, SMF = Superior Middle Frontal cortex, SV = Simple Virilizing CAH, SW = Salt-Wasting CAH, TML = Temporal Medial Lobe, WM = White Matter,
